# How Can We Enhance Adherence to Medications in Patients with Systemic Lupus Erythematosus? Results from a Qualitative Study

**DOI:** 10.3390/jcm11071857

**Published:** 2022-03-27

**Authors:** Sharzad Emamikia, Cidem Gentline, Yvonne Enman, Ioannis Parodis

**Affiliations:** 1 Division of Rheumatology, Department of Medicine Solna, Karolinska Institutet and Karolinska University Hospital, 17176 Stockholm, Sweden; cidem.gentline@regionstockholm.se (C.G.); yvonne.enman@ki.se (Y.E.); 2 Department of Rheumatology, Faculty of Medicine and Health, Örebro University, 70182 Örebro, Sweden

**Keywords:** systemic lupus erythematosus, medication adherence, compliance, patient perspective, qualitative research

## Abstract

Medication non-adherence is common among patients with systemic lupus erythematosus (SLE) and may lead to poor clinical outcomes. Our aim was to identify influenceable contributors to medication non-adherence and suggest interventions that could increase adherence. Patients with SLE from two Swedish tertiary referral centres (*n* = 205) participated in a survey assessing self-reported adherence to medications. Responses were used to select patients for qualitative interviews (*n* = 15). Verbatim interview transcripts were analysed by two researchers using content analysis methodology. The median age of the interviewees was 32 years, 87% were women, and their median SLE duration was nine years. Reasons for non-adherence were complex and multifaceted; we categorised them thematically into (i) patient-related (e.g., unintentional non-adherence due to forgetfulness or intentional non-adherence due to disbelief in medications); (ii) healthcare-related (e.g., untrustworthy relationship with the treating physician, authority fear, and poor information about the prescribed medications or the disease); (iii) medication-related (e.g., fear of side-effects); and (iv) disease-related reasons (e.g., lacking acceptance of a chronic illness or perceived disease quiescence). Interventions identified that healthcare could implement to improve patient adherence to medications included (i) increased communication between healthcare professionals and patients; (ii) patient education; (iii) accessible healthcare, preferably with the same personnel; (iv) well-coordinated transition from paediatric to adult care; (v) regularity in addressing adherence to medications; (vi) psychological support; and (vii) involvement of family members or people who are close to the patient.

## 1. Introduction

Systemic lupus erythematosus (SLE) is a multisystem, autoimmune disease that most commonly affects women during their fertile years of age. While SLE can affect all organ systems or tissues, involvement of the skin, joints, central nervous system, and kidneys are among the most frequent manifestations [1]. Given that SLE is a chronic disease, the patients are generally treated with long-term regimens or even life-long immunomodulatory or immunosuppressive medications including antimalarial agents (AMA), glucocorticoids (GCs), and conventional synthetic or biologic disease-modifying anti-rheumatic drugs [1]. Adhering to medications can be a challenge for patients with chronic diseases; non-adherence has been reported to occur frequently in patients with SLE [2] and be associated with poor treatment outcomes [3,4] and a higher likelihood of developing irreversible organ damage [5]. Proportions of patients with SLE who are non-adherent to medications range from 43% to 75% in different studies, depending on how non-adherence was assessed [2]. Various methods are used to measure the degree of adherence to medications including patient-reported tools [6,7,8,9] and measures that are designed to be filled-in by others than the patients themselves (e.g., healthcare professionals [10,11,12,13]), all primarily used for research purposes.

The reasons for intentional non-adherence are less precise than those for unintentional, the latter being commonly associated with forgetfulness. Overall, intentional non-adherence include problems related to taking medications (e.g., side-effects, inability to pay for the medications, which is more prominent in developing countries, or disagreement regarding the need for pharmacological treatment) [14]. Negative beliefs regarding medications in general or about particular medications are also likely to be associated with intentional non-adherence [15]. For example, the most common reasons for discontinuation of AMA on the patient’s own initiative include the perception of AMA not being an effective treatment and apprehension about the potential side-effects [11].

There are different ways to positively impact on the level of adherence to medications. Explaining to the patients how taking a specific drug will benefit them and communicating information about the potential side-effects of the drug have been shown to be important elements, as have providing in-depth information about the disease [16,17]. In this respect, well-informed patients may acquire more positive beliefs on medications and make grounded shared decisions together with their physician, which they are more inclined to follow [18,19,20,21,22,23].

Under the overall aim of identifying influenceable factors that contribute to medication non-adherence, the specific objective of the present study was to interview patients with SLE from the Karolinska and Örebro University Hospitals to identify such factors in the Swedish healthcare context, and thus propose interventions that could enhance adherence to medications.

## 2. Materials and Methods

### 2.1. Study Design

We performed structured qualitative interviews with individual SLE patients between August 2021 and November 2021. The patients were recruited from a larger online survey study that was conducted in 2021 and investigated the impact of different factors on medication adherence. The location of the interviews (digital or in person meetings) was decided by the interviewees in order to ensure a safe environment for the person being interviewed.

### 2.2. Patient-Reported Medication Adherence

Medication adherence was self-reported by the patients. The frequency of intake of GCs, AMA, and other medications was measured separately using the 6-item Medication Adherence Self-Report Inventory (MASRI) [7], a questionnaire that includes two parts (A and B) where part A contains specific questions on the amount of medication taken recently to prepare the person for the last follow-up question, which is a visual analogue scale (VAS) estimating the overall medication adherence during the past month from 0 (no medication intake) to 100% (full intake). The results from the VAS are used to determine the medication adherence level. Part B of the MASRI is focused on the exact timing of the medication intake; this part was not administered to the survey study participants. The MASRI (part A) has been shown to be reliable for measuring medication adherence in patients with SLE [24]. In addition, we used the Compliance Questionnaire Rheumatology (CQR) [9,25], a 19-item instrument that is more specific for rheumatic diseases and provides a comprehensive assessment of the adherence status. In the CQR, the respondent indicates the level of agreement with different statements made by patients with rheumatic disease. The CQR yields adherence levels from 0% to 100%.

### 2.3. Patient-Reported Disease Activity and Organ Damage

Disease activity was assessed with the Systemic Lupus Activity Questionnaire (SLAQ) [26,27], which captures symptoms and disease activity related to SLE in the preceding three months. The SLAQ assesses the presence and severity of flares and comprises 24 items that investigate SLE-related symptoms, yielding a symptom score from 0 to 24, depending on the presence or absence of symptoms. Higher scores indicate higher disease activity. The questionnaire ends with a VAS (global health score) that estimates the disease activity, where 0% reflects no disease activity and 100% represents the highest imaginable disease activity.

Patient-reported organ damage was estimated using the Self-Administered Brief Index of Lupus Damage (SA-BILD) instrument [28]. The SA-BILD includes 28 items covering ocular, neuropsychiatric, renal, pulmonary, cardiovascular, peripheral vascular, gastrointestinal, musculoskeletal, skin damage, premature gonadal failure, diabetes, and malignancy. Higher scores indicate greater levels of accumulated irreversible organ damage; the scale ranges from 0 to 30.

### 2.4. Selection of Patients for Qualitative Interviews

Patients from two Swedish tertiary referral centres (i.e., the Karolinska and Örebro University Hospitals) with an International Classification of Diseases (ICD) code indicating that they are diagnosed with SLE were asked to participate in a survey-based study addressing medication non-adherence. In this survey, patients were asked to indicate whether they were willing to be contacted for participation in a subsequent qualitative interview-based study. From 205 patients who completed the survey, 15 patients from the Karolinska University Hospital in Stockholm (*n* = 14) and Örebro University Hospital in Örebro (*n* = 1) were selected to be invited to participate in individual in-depth interviews for the purpose of the present study. To obtain in-depth information from a representative sample from the survey respondents, our selection of interviewees aimed at a varying degree of experience of challenges in adhering to medications (i.e., varying levels of adherence) and a similar age range and sex distribution to that of the population of patients who filled in the survey.

### 2.5. Interview Protocol

The English version of the interview protocol is presented in the online Supplementary Materials. The protocol was designed to address the main research question of the present investigation (i.e., what kind of implementations in healthcare could improve medication adherence in patients with SLE), as derived from the perspective of the interviewees. The main interview question was: “What do you think could make you take your medicine exactly as prescribed by your treating physician?”. The questions posed to the participants were short and easy to understand. The interview was initiated with questions starting with “what” and “why”, referring to a concrete event. Once those questions were answered, questions involving “how” were pursued. Issues starting with “why” and dealing with the patient’s reasons for their potential lack of medication adherence were addressed towards the end of the interview. Questions of a “probing” nature were prepared with the aim of following up on certain answers with subsequent questions, gradually leading to more comprehensive information [29].

### 2.6. Interview Procedure

All interviews but one were performed in Swedish. In that interview, the questions were posed in Swedish, but the interviewee preferred responding in English. All interviews were performed by the same investigator (SE). The interviewees were informed prior to the start of the recording that the interviews would be recorded to preclude recall bias and eliminate the risk of misinterpreting the patient, and recording started upon the patient’s approval. Verbatim transcription was performed by the same investigator who performed the interviews (SE), directly after each interview with the aim of implementing improvements in the subsequent interviews. Even though the interview guide was structured with predefined questions, it was inevitable that some parts of the interview were conducted ad hoc with the help of probing questions [30], and was thus dependent on the interaction between the interviewee and the interviewer. A pilot interview preceded the aforementioned 15 interviews as an aid to design and fine-tune the interview guide in compliance with qualitative research conduct methodology. Due to the ongoing severe acute respiratory syndrome coronavirus 2 (SARS-CoV-2) pandemic, most interviews were performed in a digital format using video conferencing services to virtually meet the interviewees (*n* = 13). One of the interviews was conducted at the inpatient care unit at the Karolinska University Hospital and another interview at the patient’s working place.

### 2.7. Analysis of Qualitative Data

Content analysis, a flexible method to analyse text data [31,32,33], was performed by two investigators (SE and CG) who independently read and reread the transcripts in detail, in order to index and compare the identified subcategories. The subcategories were derived inductively (data-driven) and were centred on types of behaviours (i.e., reasons for not displaying adequate adherence to medications). The role of subcategories was to increase the granularity of data collected, ensuring a broad and inclusive process that would help comprehend the data as much as possible. Categories with substantial similarities were later unified to create key themes. Depending on how the patients described their behaviour and reasons for not adhering to medications, we stratified the reasons into intentional and unintentional based on whether the patient had actively decided to be non-adherent or not, respectively.

Data collection continued until the discussion material had reached saturation (i.e., until no additional data were identified by analysing the transcribed interviews) [34,35]. Data saturation was based on identification of new themes and categories while analysing the transcripts (i.e., inductive thematic saturation) [36]. We considered the data saturated when results from the last five interviewees did not add novel reasons for not adhering to medications as prescribed by the treating physician and when no new interventions for improving medication adherence were mentioned during the interviews. Potential discrepancies between the two investigators (SE, CG) were resolved by reaching a consensus together with a third investigator (IP), who is a rheumatologist.

### 2.8. Patient and Public Involvement

The research question of this study was developed in collaboration with a patient research partner (YE), based on shared priority to investigate adherence to medications. The patient research partner was involved from the initial stage of the research process and throughout the development and conduct of the study.

### 2.9. Ethics

The study design and conduct complied with the ethical principles of the Declaration of Helsinki. The protocol of the study was approved by the Swedish Ethical Review Authority on 1 April 2021 (reference number: 2021-00662). Written informed consent was obtained from all study participants prior to enrolment.

## 3. Results

### 3.1. Patients

All study participants were adults (age range: 20–68 years) and most of the participants were women (*n* = 13; 87%). In addition to these participants, two patients were invited to participate but declined, one who was a public figure and worried that their identity might be revealed and one without providing any specific reason. Patient characteristics including patient-reported disease activity and organ damage are presented in Table 1 (*n* = 14; data were missing in one patient). One of the participants had experienced a severe flare within three months prior to the time of filling in the initial survey. Adherence levels based on inter alia responses to the MASRI and CQR are detailed in Table 2. The patients in the original survey study (*n* = 205; age range: 20–87 years) had a median disease duration of 12 (5–22) years, and 86% among them were women.

### 3.2. Barriers to Medication Adherence

We identified four main themes that explained medication non-adherence in this cohort, which are demonstrated below along with selected illustrative quotations from the interviewees translated from Swedish to English.

#### 3.2.1. Patient-Related Barriers

In total, the proportion of patients who expressed patient-related barriers at least once was 93.3% (*n* = 14). Patients expressed that being diagnosed with a chronic disease at a young age was important to account for (*n* = 5; 33.3%), as were their professions (*n* = 8; 53.3%). Unintentional non-adherence was mainly caused by forgetfulness (e.g., due to interruptions in the patient’s routines) (*n* = 5; 33.3%).

*I know if I’m traveling, it’s easy to forget. (Patient 15)*

*And sometimes I forget why it is a good idea [to take the medications], but I think the practicalities of life sometimes and all the things you have going through in your head […] get in the way… (Patient 08)*

*I’m in too much of a hurry. There’s no time. It takes time to prepare a sandwich. This is the kind of thing that is done as the last... last priority. (Patient 12)*

In this respect, having to keep track of multiple pills and multiple or varying timepoints for the intake were mentioned as barriers (*n* = 5; 33.3%). Unwillingness to take multiple medications (*n* = 3; 20.0%) was discussed. Factors related to the administration of the medications also seemed to cause irregular adherence (e.g., having to split the pill or having to take varying doses of a medication each day) (*n* = 1; 6.7%). The size and taste of the pills hindered some patients in taking their medications (*n* = 2; 13.3%), while some other patients experienced a resistance at a mental level when having to inject themselves with a medication (*n* = 1; 6.7%).

*I can’t stand fiddling with all the pills in the morning. (Patient 12)*

*You don’t want to feel so sick; you skip some. Sometimes you take a few and sometimes you don’t take anything. (Patient 09)*

*I don’t know if there are too many, it’s just that it’s too much to stand and fiddle with. I have six medicines and stand and push them out, pick them out, it sounds silly when I say it like this. I can’t stand it, it’s so hard. I don’t know why it’s so hard. (Patient 12)*

*When I was younger, I ate a lot of pills. Some days it felt like you were taking ten pills for breakfast. It’s too much. I felt nauseous, some of the medications made me nauseous. And then you know you’re going to do that for the rest of your life, feeling bad about medications that are supposed to make you feel good. (Patient 10)*

Moreover, various practicalities were mentioned as potential causes of non-adherence (e.g., difficulties in obtaining medications from the pharmacy) (*n* = 1; 6.7%) or because of other specific circumstances (e.g., due to the SARS-CoV-2 pandemic) (*n* = 1; 6.7%).

Several patients raised beliefs indicating that they themselves or family members were sceptical towards medications that they were prescribed, which negatively impacted on their adherence (*n* = 5; 33.3%). The mother of one of the interviewees wanted the treatment to also include herbal remedies. One of them mentioned that the scepticism was due to a previous adverse event related to an intravenous treatment, and because the rheumatologist was too focused on the present condition rather than the prospective aspects of the disease. Another patient mentioned the importance that religion had in their family.

*I grew up in a very religious [religion specified] home, and in such a home you think you don’t need medicines because your body knows how to treat itself. (Patient 07)*

Scepticism could also be expressed in general terms.

*I can sometimes think, do I need all those medications? How do we know that this particular medication is helping me, or how do we know that this medication I am given now is helping me? Why am I given so much? Why haven’t we sticked with some of the medications? (Patient 10)*

#### 3.2.2. Healthcare-Related Barriers

The overall percentage of patients who expressed barriers related to the healthcare system at least once was 73.3% (*n* = 11). The relationship with the treating physician was central according to most interviewees (*n* = 11; 73.3%). An untrustworthy relationship with the physician could be caused by the feeling of overall inaccessibility to healthcare providers (*n* = 2; 13.3%), not being listened to (*n* = 4; 26.7%), and application of treatment strategies based on group-level evidence rather than person-centred approaches (*n* = 3; 20.0%).

*My doctor didn’t really listen to me that much and it was more of a generalisation every time I saw my doctor. (Patient 05)*

*They basically prescribe the same thing to all SLE patients. (Patient 07)*

Some patients felt like they were seen as “an SLE diagnosis” rather than “an individual behind the SLE diagnosis” (*n* = 2; 13.3%).

*There is a normal person behind the civic registration number. (Patient 04)*

Additionally, the impression of not being believed as a patient when telling their physician about symptoms (*n* = 2; 13.3%) and meeting different healthcare personnel from visit to visit (*n* = 2; 13.3%) seemed to negatively impact on adherence.

*I’m not a guinea pig… (Patient 01)*

There was an interest in knowing the indications of the medications in more detail (i.e., whether the medication prevents symptoms or treats the disease) (*n* = 2; 13.3%). Some expressed the desire to receive more information about the potential side-effects and mechanisms of action of the drugs (*n* = 4; 26.7%).

*And then I go home and do research myself and find out what the medication does, what the side-effects are. Because they don’t always tell you about the side-effects either. It comes as a shock. (Patient 09)*

Some patients had experienced that physicians considered test results more important than the patients’ experience of health-related quality of life (HRQoL), which also negatively affected the trust towards healthcare and, in turn, adherence to medications (*n* = 3; 20.0%).

The interviewees expressed concerns resulting from lack of information about whether consumption of alcohol or certain kinds of food could interact with the medication, or whether fasting had an impact, which led to not taking their medication at certain timepoints (*n* = 3; 20.0%).

Several patients (*n* = 6; 40.0%) experienced a power imbalance between the patient and the physician. This power imbalance could cause non-adherence due to fear of authority, resulting in not finding the courage to question the physician (*n* = 1; 6.7%), feeling embarrassed to pose questions about the medications (*n* = 1; 6.7%), or feeling that they take control when not adhering (*n* = 1; 6.7%).

*It’s a bit of a position of power and you still need help from that person, and it can be hard to speak out. (Patient 05)*

#### 3.2.3. Medication-Related Barriers

In total, the percentage of patients who expressed medication-related barriers at least once was 66.7% (*n* = 10). Among those, intentional non-adherence was common, with the belief that the prescribed medication might cause side-effects being the most common reason (*n* = 10; 66.7%).

*Cortisone, I’ve always been against it… (Patient 01)*

Some of the patients (*n* = 8; 53.3%) were worried about potential side-effects that could occur in the future, and other patients (*n* = 7; 46.7%) were worried that side-effects they had previously experienced from the prescribed medication would emerge again. Patients expressed concerns that the medications might result in a shorter lifespan (*n* = 1; 6.7%), weight gain (*n* = 1; 6.7%), nausea (*n* = 1; 6.7%), and skin dyspigmentation (*n* = 2; 13.3%). Some interviewees also raised apprehensions about potential negative effects on the kidneys (*n* = 1; 6.7%) and eyes (*n* = 2; 13.3%), and that the prescribed medication might cause osteoporosis (*n* = 1; 6.7%) and/or diabetes (*n* = 1; 6.7%).

For some interviewees, intentional non-adherence could be a consequence of poor information about their prescribed medications (*n* = 8; 53.3%).

*It’s often you feel, what am I putting in me? (Patient 10)*

The perspective of time seemed to be important (e.g., information about how long the medication is planned to last), especially when a substantial difference in symptoms was not experienced after commencement of a new medication (*n* = 2; 13.3%). In such cases, non-adherence was the result of testing the efficacy of the medication on the patient’s own initiative (i.e., to see whether there was a difference in symptoms when not taking the medication).

*I don’t notice any difference if I skip the medication for a day or two. (Patient 04)*

#### 3.2.4. Disease-Related Barriers

The overall percentage of patients expressing disease-related barriers at least once was 53.3% (*n* = 8). Some patients perceived the severity of their disease as mild, which negatively affected their adherence to medications (*n* = 6; 40.0%).

*I don’t have very serious symptoms. It doesn’t matter much if I don’t take the medication. (Patient 05)*

Some patients believed that it was difficult to talk to other people about their disease since the disease was not always tangible, which could result in neglect (*n* = 1; 6.7%). Along the same lines, patients were less motivated to take their medications when feeling well because no symptoms reminded them of the need to take their medications (*n* = 6; 40.0%).

*I have stopped when I have felt good. I felt like I didn’t need them anymore. (Patient 07)*

*Usually when I’m okay. I forget that I am sick and need to take the medication. (Patient 06)*

With regard to disease manifestations and/or comorbid conditions, depression was mentioned as a reason for non-adherence (*n* = 1; 6.7%) that occurred previously in the patient’s life but was not manifest at the time of the interview. Finally, acceptance of a chronic illness was difficult for some patients (*n* = 5; 33.3%), partly because in several cases the disease is not visible to others and therefore difficult to talk about, or because taking multiple medications would make them feel that they are sick.

*I guess it is that you don’t feel you are sick. You have side-effects. I don’t want to feel sick; I want to be a healthy person. (Patient 12)*

*I hate feeling sick… (Patient 01)*

*I was still very sick at that time…now that I’m healthy. Or well, healthy… (Patient 07)*

### 3.3. Positive Impact on Medication Adherence

Patients explained that they also had several reasons for taking their medications as prescribed. Many experienced that they felt balanced regarding their well-being when they took their medications (*n* = 7; 46.7%), they avoided flares (*n* = 6; 40.0%), they had less pain and more energy (*n* = 5; 33.3%), and they could be physically active and able to work (*n* = 3; 20.0%). Several patients (*n* = 6; 40.0%) described the use of pill organisers as an important tool for maintaining a high adherence level, despite some resistance in accepting them.

*I feel so old. Only old ladies have such pill organisers. (Patient 12)*

### 3.4. The Impact of Shared Decision-Making

In the interviews, the patients talked about their view on shared decision-making. Notably, several patients in our study (*n* = 6; 40.0%) had the impression that their involvement in therapeutic decision-making would not affect their adherence to medications.

*I have no medical knowledge of these medications. I have to trust their professionalism and knowledge. I can’t do more than that. My doctor is a specialist in the field. (Patient 12)*

*How can you be involved in something you don’t know anything about? […] I don’t know anything about these medications. It’s the doctor who’s a specialist, I listen and test. If it goes well, I’ll be fine. (Patient 03)*

*I’m not medically trained. […] You accept what you get. (Patient 14)*

*I probably leave it to them… they know what is best for me. (Patient 08)*

One patient felt that she was not given the opportunity to be involved, even though she did not believe that shared decision-making would impact on her adherence (*n* = 1; 6.7%).

*God no [reaction on whether the patient felt involved in the decision-making for the prescribed medications]. I don’t think it would make any big difference quite honestly since they [the rheumatologists] know what they are prescribing, and they basically prescribe the same thing to all SLE patients. The names of the drugs may differ slightly, but they are still immunosuppressants and cortisone. So, I don’t think it would make any big difference. (Patient 07)*

One patient even felt that more responsibility was put on them through shared decision-making.

*Rather, I feel that I perhaps have had a little too much participation and I have been able to decide, but I did not really receive any recommendations or advice, which I would have liked. (Patient 05)*

### 3.5. Facilitators for Improving Medication Adherence

Based on the thematic analysis and direct suggestions by the interviewees, the following distinct interventions were identified as potential strategies for enhancing medication adherence.

#### 3.5.1. Increased Communication

Patients called for comprehensive information from their treating physicians about the prescribed medications (e.g., pharmacodynamics and pharmacovigilance). It was a desire that this information is person-centred and accounts for the individual situation and circumstances of the patient. Such information should ideally be provided on a regular basis.

*Maybe it’s because you don’t know what’s in that medicine that is supposed to make you feel better. (Patient 04)*

*Or, above all, tell us about late complications if you don’t take them [the medications]. Not as a threat. If you don’t do this, this will happen. Take the time to tell us what can happen. Or what the risk is if you do not treat [the disease]. (Patient 12)*

Prolonged visits and interactive encounters with the treating physicians might reduce the stressful impression during a visit when patients sometimes feel that they cannot ask questions or are unable to absorb the information that is provided to them, especially when abbreviations are used in the communication, as pointed out by one interviewee.

*There is such a short time for these appointments with the doctors that... it doesn’t feel like you’re getting an answer to all questions. (Patient 10)*

*There are a lot of things you miss when you go to your visit because you are nervous, and you need to take notes. (Patient 02)*

#### 3.5.2. Patient Education

Personalised information could be given in different formats (e.g., in writing). Additionally, congresses specifically targeted to patients could be organised, aiming for patient education about the disease of SLE and common medications used to treat it.

*They told me “You should be happy if you survive until you are an adult” and “Don’t expect to be able to have a job, you will be retired early and you will not have any children”. (Patient 04)*

Personalised adjustment of medications accounting for the patient’s lifestyle was suggested (e.g., if a patient has problems taking their medication at certain timepoints, rearrangement of the treatment regimen could be attempted). Adjustment of the communication forms accounting for the patient’s lifestyle also emerged as a desirable action (e.g., if the patient is an athlete or exercises in a regular and systematic manner, a focus on physical dimensions of how the disease afflicts the body might be useful).

#### 3.5.3. Accessible Healthcare

Easily accessible healthcare services for questions also emerged as a desire. Digital options could be provided to eliminate frustration due to difficulties in reaching the healthcare providers within a reasonable time. Another suggestion from a patient was to develop an application where patients monitor their symptoms and are able to see how medication non-adherence is associated with flares, similar to one created by and for patients with rheumatoid arthritis, where one of the multiple objectives was to increase the understanding of the impact of adherence [37]. Furthermore, meeting the same treating physician and nurse at all visits (i.e., personnel that becomes familiar to the patient and knows the patient’s medical history) appeared to be valuable for the patients.

*All these years when I’ve been able to see the same nurses in the same departments. You can tell it gives a lot. The greetings. Several nurses have seen me and asked “how are you?” Then back in action. They don’t make any big deal of it. Let’s go, let’s go. You’ll be here for 2–3 days and you’ll be back on track. It gives a feeling of safety. (Patient 01)*

#### 3.5.4. Structured Transition from Paediatric to Adult Care

In our study population, five of the interviewees had childhood-onset SLE. Special support may be of particular importance in certain phases of the disease course, or at certain arrangements; a smooth and structured transition from paediatric to adult care was referred to as an example. Several patients experienced challenges to accept the changes in their encounter with the healthcare services posed by this transition. This was described to have inevitable consequences regarding trust issues and, in turn, adherence to therapy.

*When I got SLE, it was my parents who read through it [leaflet] because I probably didn’t understand much. […] My parents used to remind me and gave me the medication every morning. (Patient 13)*

*When I went to the “Children’s’ Hospital”, back then I felt like I got a lot out of it. It was also when I was sickest. I received a lot of help and had a close contact with my doctor. […] It was easy to get in touch with them if there was anything. But then... Now […] I don’t get the help I feel I need. It’s quite difficult to contact the doctor and […] book an appointment when something is wrong. (Patient 05)*

*I’ve been taking the medications for so long that I don’t feel like I’ve had an adult life as a free person, so I’ve never had any other life before. (Patient 14)*

#### 3.5.5. Medication Reconciliation

A straightforward communication was also suggested for the process of medication reconciliation, which occasionally may be complicated. Medication reconciliation could be completed in different formats (e.g., by directly asking the patient whether they take the prescribed medications, or by asking indirectly via electronic questionnaires at the outpatient clinic before the visit, or through the Swedish online healthcare guide service).

*This would mean to me that they follow up on my illness and I would be happy to receive such a question. (Patient 02)*

*At every encounter with medical staff, because then it also becomes a reminder […] it would mean to me that they follow up the patient and keep track. (Patient 07)*

#### 3.5.6. Psychosocial Support

Psychosocial support aiming for acceptance of the diagnosis emerged as an important need. The chronic nature of the disease appeared to necessitate supportive or therapeutic counselling, especially during the early stages after the diagnosis is made.

*I think that you need a contact with a social counsellor… people who are sick with diseases that are difficult to live with need more mental support than what is offered today. (Patient 03)*

*I was never offered to talk to anyone. Maybe you should offer all patients to talk to someone, then if this person is a psychologist or a nurse, it doesn’t matter to me. (Patient 07)*

Patients call for a focus on aspects that impact on physical and mental HRQoL.

*[I want to] build up some muscle, and I feel like nothing is happening. I don’t get any support from the healthcare system... The important thing for them is that I’m “healthy” and the blood tests look good. They don’t mind about my quality of life. (Patient 11)*

*They are very happy to check test results. They can say “your blood tests look great! I’m glad to see these results!” Ok, but what are we going to do about those days when I didn’t feel so good? Then nothing happens. (Patient 10)*

#### 3.5.7. Engaging Family Members

Some patients mentioned how people who were close to them could have an impact on them. One example was sceptical family members who weakened the belief that medications can treat diseases.

*So that you kind of find out what the family’s religion and perceptions of medicine are so that you don’t just throw the patient into something that the patient won’t follow later. (Patient 07)*

*Relatives know best. You’re a stubborn man or woman, so they [the rheumatologists] might have to approach you from somewhere else [reach out to relatives]. (Patient 01)*

*I have a mother who is very sceptical and puts pressure on me all the time. (Patient 11)*

Thus, it emerged as an action of particular importance to involve people who are close to the patient in the information about the suggested or the planned treatment regimen, as well as involve them in broader aspects of patient education.

## 4. Discussion

Through in-depth interviews, we aimed at understanding the perspectives of SLE patients on factors that contribute to medication non-adherence and identifying suitable interventions or actions that could improve adherence if implemented in healthcare. Patient education at multiple levels emerged as an imperative need; patients desired more in-depth information by the healthcare personnel compared with what they currently receive, including information about disease features, blood, and urine test results, and ongoing or suggested treatment regimens. Prolonged visits with the treating physician to ensure sufficient time to address all aspects of the disease and the current condition was requested by most patients. The importance of educating the patient and communicating information in a manner that ensures that the patient feels confident has been discussed in previous research [38,39]. Several patients in our study were reluctant towards involvement in therapeutic decision-making, while many patients desired more information from their treating physician about their disease and their medications. As a matter of course, unwillingness to participate in decisions and a feeling of insufficient knowledge about the suggested medications may be related to each other in a causal manner, with the former emerging as a consequence of the latter [40]. Regular discussions about adherence to medications also emerged as a suitable action for the identification of non-adherence patterns, and when unintentional non-adherence is detected, practical advice on how to manage to effectuate regularity in taking the medications should be provided. In some cases, psychological support may be needed; it is important to establish strategies for identifying such needs and take action to provide this resource.

Overall, our findings concurred with suggestions for interventions against medication non-adherence derived from previous studies involving patients with SLE [2,41,42] and other chronic diseases [43]. Several previous studies have described methods for enhancing adherence to medications in patients with SLE [16,17], some of which were qualitative studies (e.g., from Jamaica, Portugal, the United Kingdom (UK), and the United States (US)) [18,19,20,21,22,23], while others explored and evaluated specific interventions in patient groups [44,45,46,47]. Improved communication between the healthcare and patients along with increased information about the rationale for taking the medication as well as less complicated medication regimens improved adherence in an American study [16]. Another study from New Zealand [17] provided implications that addressing patients’ concerns about side-effects may improve the relationship between the treating physician and the patient and thereby improve adherence. As in our study, results from other qualitative interview studies [18,19,20,21,22,23] suggested that improvements in communication between physicians and patients, making medications affordable and available, increasing the patients’ knowledge about the disease and therapeutic options, challenging the patients’ beliefs regarding medication effectiveness with well-documented evidence as well as facilitating access to healthcare, all are crucial factors that can be expected to contribute to increasing medication adherence.

The applicability and effectiveness of various interventions should also be seen from the perspective of the cultural background of the respective patient population. Targeted nursing including detailed patient-specific solutions that involved the patient’s daily life has been shown to be effective in improving medication adherence in Chinese patients [44]. In that study, the patients were followed up for 20 months and received support in various aspects related to understanding the disease and its prognosis, accounting for the patient’s socioeconomic background and psychological health. The patients received information about the importance of appropriate treatment, diet, prevention of infections, and other aspects [44]. However, not all studies have shown that increased communication improves adherence. A study from the US [45] examined the usefulness of cellular text messaging for improving the adherence to hydroxychloroquine (HCQ) in adolescent SLE patients, but despite this type of individualised communication, the repetitive messages did not influence the patients’ degree of adherence. In this respect, it is reasonable to postulate that the means of communication may matter. Another US study showed that routine testing of the blood levels of HCQ improved adherence over time, from 56% to 80% adherent patients [46]. A study from India evaluated the role of a clinical pharmacist in terms of individualised counselling regarding the disease, medications, and lifestyle modifications using pre-developed written educational material [47]; counselling performed monthly and three times in total resulted in improved adherence to medication.

Interestingly, several patients in our study referred to their professions while being interviewed. Some patients worked within healthcare themselves, others were athletes; irrespective of their profession, patients related several of their responses to their occupation, which raises awareness of the importance of taking the patient’s profession and background into account in order to provide individualised information. Another aspect that pointed to the importance of person-centred approaches was that the patient’s age was important for the patients, both the current age and the age when the diagnosis was made, with a younger age in both cases being coupled with a lower level of acceptance of illness and a lower degree of motivation to adhere to regularity in taking medications. Therefore, these patient subgroups may need more attentive contact with their physicians and/or other health professionals involved in their care, and a higher level of support. The transition from paediatric to adult care appeared to be challenging, in conformity with what has been shown in other studies [48,49], especially when the patient had a strong bond to the paediatrician. All the above set a strong motive for the healthcare and treating physicians to tailor the communication, the information about the disease and therapies, and the surveillance strategies to the patient’s individual background.

Some patients mentioned that multiple medications and complicated schedules of varying daily doses constituted a reason for impaired adherence. Polypharmacy, defined as five or more concurrent medications [50], has been documented as a factor contributing to non-adherence in previous studies [13,51], collectively suggesting that regular and thorough medication reconciliation and, when possible, attempts to decrease the total amount of daily administrations may help improve adherence to medications.

Suffering from depression has been shown in previous research to cause poor adherence to medications in patients with SLE [4,52], which was also detected in our interviews. Along with symptomatically treating depression, more attentive follow-up and support directed to improve adherence in this patient subgroup might prove helpful. Depression can influence forgetfulness [4] and has been described as an independent risk factor for non-adherence [13]. Considering that a substantial proportion of patients with SLE develop anxiety or depression [1], which is often resistant to therapy for SLE [53], this patient subgroup may be of particular importance to consider in the allocation of healthcare resources to actions against medication non-adherence.

In patients with SLE, the use of AMA has been associated with a wide variety of beneficial effects [54,55], including favourable associations with biological disease properties such as lower levels of B cell activating factor (BAFF) [56] and favourable effects on HRQoL [57]; this drug class constitutes the cornerstone of SLE therapy. During our interviews, it appeared cumbersome to retrieve the correct daily dose of HCQ when a different number of pills was to be taken on different days of the week according to the prescription. One patient called for more flexible dosage schedules or an easy way to divide the pill instead of varying dosages each day. This issue has also been addressed in a large study from the US comprising 3127 patients with SLE, which demonstrated that a substantially higher proportion of patients taking different doses every day reported that they forgot or mistook the correct dose (32%) compared with patients who were on the same dose every day [58]. Thus, prescriptions that are easy to follow may ascertain that the patient will follow them to a larger extent. While this notion may appear intuitive, complicated dosage schedules are often used to adjust for the patient’s weight, raising actionable awareness of the need for a reduction of the degree of complexity when designing therapeutic regimens.

Financial costs of medications have been identified as potential barriers to medication adherence in patients with SLE in a study from the US [21], as well as in documentations from developing countries [19]. Along these lines, one interviewee mentioned that there had been times when the patient did not pick up a prescription for SLE from the pharmacy due to the price of the medication to prioritise other medications prescribed for other diseases. This affirmation by the patient is of interest considering the Swedish context, where costs for prescribed medications exceeding a yearly amount of 2350 SEK (equivalent to USD 262, based on the current exchange rate), underlying that therapeutic decision-making and patient education should also account for comorbidities and raising actionable awareness of the need for the identification of impecuniousness in certain cases and allocation of resources to ensure access to the prescribed medications.

Support from people close to the patient was mentioned by some study participants to be vital. This suggests that information and education should not only be provided to the patients, but also to family members or trusted people from the patient’s environment. In this regard, a previous study showed that a clinical pharmacist could have an important role in counselling family members and providing them with information about the prescribed medications [47]. Last, but not least, not only the socioeconomic background, but also values important to the patient should be accounted for in therapeutic decisions and in patient education to ensure a holistic person-centred strategy against medication non-adherence. Such values may include the religious beliefs of the patient or family members, which was described by one of the interviewees to be of particular importance. Religious and spiritual beliefs have also been described to impact on medication adherence in previous research [19], especially when such beliefs specifically address or interfere with the use of medications or the time of the day when medication intake is scheduled. The same study [19] identified perceived mild severity of the disease as one of the reasons for medication non-adherence; some patients tended to take their medications only when experiencing symptoms, which is in compliance with the findings in the present study. However, high disease activity and disease flares may also result in difficulties with adhering to medications, as shown in another study [4]. While these discrepancies across studies may give the impression of being conflicting, a reasonable explanation might be that the level of disease activity impacts on how adherence is affected (e.g., with low-grade activity acting as a “reminder” and high-grade activity being a negative contributor), as do cultural and socioeconomic facets.

### 4.1. Limitations and Strengths

The small study population may be considered a limitation. However, this study was designed to be a qualitative thematic analysis of interview content and data collection from individual interviews that continued until the material had reached saturation (i.e., until no additional content or patterns were identified during the analysis). Thus, a larger number of interviewees was deemed excessive and even inappropriate, as it might result in a cumbersome dataset to analyse while not adding informative data. The study was not designed to prospectively assess the impact of the identified potential interventions on SLE patients’ adherence to medications. However, the hypotheses generated herein warrant prospective investigation in future studies. Moreover, statistical determination of associations between hypotheses and data was beyond the scope of this study; thus, the analysis of the interviews was not assisted by statistics software and the presentation and discussion of our findings was mainly descriptive.

Disease duration was not considered when patients were asked to participate in the interviews. Our study participants’ median disease duration of nine years was shorter than that of the respondents to the initial survey-based study (12 years). Shorter disease duration has been associated with adherence difficulties in a study comprising 834 patients with SLE [4]. Thus, since we aimed to mainly interview patients with adherence difficulties, our selection strategy may intrinsically have resulted in a shorter median disease duration for the interviewees compared with the survey respondents.

A major strength of our study was that the interviews were performed by a researcher who did not belong to the clinic personnel and with whom the study participants therefore had no prior acquaintance (SE). An additional advantage of this was that the interviewer had no interference in the therapeutic decision-making, thus ensuring objectivity during the interviews. This made the interviewees comfortable with sharing in-depth thoughts and opinions. In a few instances, the interviewees requested assurance that their treating rheumatologist would not be made aware of their responses. However, two researchers with different backgrounds (master in biomedicine, SE; resident in rheumatology, CG) were involved in the analysis of data to ensure that the analysis and subsequent interpretation of results were not dependent on one individual investigator or biased by the view of a specific profession.

### 4.2. Clinical Relevance

Medication non-adherence may have detrimental effects on patient safety, especially when poor adherence is not identified, resulting in mistaken treatment evaluations. This may in turn lead to unnecessary dose increases or changes in the therapeutic regimens, potentially resulting in additional morbidity and impairments of the patients’ HRQoL. Towards the goal of improving SLE patients’ adherence to medications, the first and most important step is to systematically seek modifiable factors that negatively impact on adherence at the level of the individual patient, and thereafter, together with the patient, couple these factors with person-centred interventions against non-adherence.

## 5. Conclusions

We identified several potential interventions for improving medication adherence in patients with SLE. Our findings suggest that patients should be educated by obtaining complete written and oral information about the medications that they are recommended or prescribed and about the disease that they are diagnosed with, in order to increase their knowledge and deepen their understanding of the rationale for taking the medications. Healthcare professionals should account for the patients’ HRQoL, religious beliefs, and professions, and include family members in the care process. Professional mental support should be offered to help the patients accept the diagnosis of a chronic disease and the need for treatment. Direct and empathic medication reconciliation on multiple occasions emerged as a desire from the patients. Finally, digital options for providing information and for communication between healthcare providers and patients also emerged as a potential facilitator. It is warranted to apply these interventions prospectively to an SLE patient population to examine the effectiveness that these strategies may have on improving medication adherence.

## Figures and Tables

**Table 1:** Patient characteristics. Data are presented as median and interquartile range. SLAQ and SA-BILD scores were missing for one patient.

Age (years); median (IQR)	32 (27–50)
Country of birth; *n* (%)	
Sweden	7 (46.7)
Other	8 (53.3)
Living alone; *n* (%)	6 (40.0)
Highest education level; *n* (%)	
University	11 (73.3)
High school	4 (26.7)
Employment status; *n* (%)	
Full time	9 (60.0)
Part time	5 (33.3)
Retired	1 (6.7)
Disease duration (years); median (IQR)	9 (5–20)
SLAQ Symptom Score *; median (IQR)	10 (6.75–15.25)
SLAQ Global Health Score ^†^; median (IQR)	35 (20–65)
SA-BILD Total Score ^‡^; median (IQR)	0 (0–1.25)

IQR: Interquartile range; SLAQ: Systemic Lupus Activity Questionnaire; SA-BILD: Self-Administered Brief Index of Lupus Damage. * SLAQ Symptom Score ranges from 0 to 24. ^†^ SLAQ Global Health Sore ranges from 0 (no disease activity) to 100 (maximum disease activity). ^‡^ SA-BILD Total Score ranges from 0 to 30.

**Table 2:** Adherence levels in the fifteen interviewees.

Patient	Polypharmacy (i.e., ≥Five Medications) (Y/N)	Prescribed Medications	Overall Medication Adherence According to:			Intentional Non-adherence (Y/N)
			MASRI (0–100%)	CQR (0–100%)	Direct Question (Y/N) *	
1	N	PRED	96	61	N	Y
		HCQ	96			
		AZA	96			
2	Y	PRED	100	77	Y	NA
		HCQ	100			
		MTX (pills)	100			
3	N	PRED	100	81	Y	NA
4	N	HCQ	90	58	N	Y
5	N	HCQ	100	54	N	Y
6	N	PRED	70	65	N	N
		HCQ	70			
		AZA	69			
7	N	PRED	67	47	N	Y
		HCQ	50			
		AZA	20			
8	N	HCQ	88	72	N	N
9	N	PRED	95	74	N	Y
		HCQ	96			
		MMF RTX (iv)	99			
10	N	PRED	100	66	N	N
		HCQ	100			
		AZA BEL (sc)	80			
11	Y	PRED	96	54	N	N
		HCQ	100			
		MMF RTX	98			
12	N	PRED	89	58	N	N
		HCQ	92			
13	N	PRED	100	84	Y	NA
		CYS	100			
14	N	HCQ	100	74	Y	NA
		MTX (pills)	100			
15	Y	PRED	90	67	N	N
		HCQ	90			
		MMF	80			

MASRI: Medications Adherence Self Report Inventory; CQR: Compliance Questionnaire Rheumatology; Y/N: yes/no; NA: not applicable; iv: intravenous; sc: subcutaneous; PRED: prednisolone; HCQ: hydroxychloroquine; AZA: azathioprine; MTX: methotrexate; MMF: mycophenolate mofetil; RTX: rituximab; BEL: belimumab; CYS: cyclosporine. * Y = adherence assent to direct question; *n* = non-adherence assent to direct question.

## Data Availability

The data presented in this study are available on request from the corresponding author. The data are not publicly available due to being interview transcripts.

## Supplementary Materials

The following supporting information can be downloaded at: https://www.mdpi.com/article/10.3390/jcm11071857/s1, Supplementary materials: Interview schedule.
