# Supplementary material for: How Can We Enhance Adherence to Medications in Patients with Systemic Lupus Erythematosus? Results from a Qualitative Study
Source: J Clin Med. 2022 Mar 27;11(7):1857. doi: 10.3390/jcm11071857 (PMC8999748; doi:10.3390/jcm11071857)
Supplement: Supplementary file 1 [file jcm-11-01857-s001.zip › jcm-1599086-supplementary.pdf]

INTERVIEW SCHEDULE

The interviews were performed in Swedish.

GENERAL ISSUES

INTRODUCING QUESTION ON SOMETHING CONCRETE

What do you think about when I say, “medication adherence”? That is “to take your medicines exactly as prescribed”. In this case, medications for SLE.

INDIRECT QUESTIONS

Why do you think some people don't take their SLE medications exactly as prescribed by the doctor?

Do you think those people know what the medications are used for and the reasons why they have been prescribed?

LACK OF MEDICATION ADHERENCE

SPECIFYING QUESTIONS

What are your thoughts on the importance of taking your medicines regularly?

Have you experienced any difficulties taking your medicines for SLE regularly?

DIRECT QUESTIONS

Could you describe what the mindset is when you decide not to take your medicines as prescribed?

What is crucial for you when you decide to not take your medicines for SLE?

What do you think is the main reason for not taking your SLE medications just as prescribed by your doctor?

How do you feel afterwards? When you look back on certain times or days when you have not taken your medicines for SLE, how does it affect you today and how do you view your decisions to not take your medications for SLE?

What would you say you gain by taking your medicines for SLE?

Do you feel that you understand why the medications you take have been prescribed and what they are for?

Do you think you lose something by taking your medicines for SLE?

SPECIFYING QUESTION

On the days when you take your medication just as prescribed, what do you think brings you to that decision?

PROBING QUESTION

Could you give more examples?

RELATIONSHIP WITH HEALTHCARE

DIRECT QUESTION

What would you say that contact with healthcare has meant to you?

SPECIFYING QUESTIONS

How do you experience the communication between you and the healthcare professional?

When you think about your hospital visits, could the medical staff have communicated differently?

FOLLOW UP QUESTION

If yes, in what way?

SPECIFYING QUESTION

Do you feel that you could participate in the decision-making for the medications that you were prescribed?

DIRECT QUESTION

Do you have any suggestions for improvement in healthcare services?

INTERVENTIONS TO IMPROVE MEDICATION ADHERENCE

STRUCTURING QUESTION

What do you think could make you take your prescribed medications?

DIRECT QUESTIONS

In what way do you think these issues should be addressed except for research purposes such as in this case?

How important would it be for you if someone from the healthcare system discussed these issues with you?

Has anyone from the healthcare system ever asked you if you are taking your prescribed medications? If not, when would you have desired to be asked about medication adherence?

QUESTIONS THROUGHOUT THE INTERVIEW

PROBING QUESTION

Could you elaborate on what you’re describing?

INTERPRETING QUESTION

Do you mean that…?
